# INFLUENZA B-ASSOCIATED HELLP SYNDROME COMPLICATING PREGNANCY: A CASE REPORT

**DOI:** 10.51894/001c.122910

**Published:** 2024-08-31

**Authors:** Calley Gober

**Affiliations:** 1 OBGYN Trinity Health Muskegon

## 43

### INTRODUCTION

HELLP syndrome is a rare but serious pregnancy complication, occurring in 0.2-0.9% of all pregnancies and 10-20% of patients with pre-eclampsia with severe features. Typical presentation includes right upper quadrant abdominal pain, nausea, vomiting, and malaise. Hypertension is often reported, though up to 20% of patients are normotensive. The pathogenesis of HELLP is believed to be due to decreased uterine blood flow, leading to placental ischemia and oxidative stress. While some case reports have postulated that oxidative stress in other inflammatory processes (e.g., COVID) induces HELLP, the authors have found few reports of influenza and HELLP.

### CASE DESCRIPTION

A 28-year-old, G3P2, at 28 weeks’ gestation, presented with severe abdominal pain and contractions every 1 minute. At a recent ER visit for a sore throat, she was diagnosed with Influenza B and discharged on Tamiflu. Her labs revealed coagulopathy with INR 1.7, prothrombin time 18.5, and thrombocytopenia (platelets 132). Initial liver enzymes were elevated (AST 1159, ALT 715). She was started on betamethasone and IV Magnesium for fetal neuroprotection and suspected HELLP syndrome. She consented to urgent repeat C-section and blood products. TXA (1g) and 1 unit of fresh frozen plasma were given, and the procedure was performed under general anesthesia. Her surgery was largely uncomplicated; QBL 754mL. Apgar’s were 6 (1 minute) and 9 (5 minutes). The neonate was intubated at 45 minutes of life and transferred to the NICU. The patient’s liver enzymes peaked (AST 6012, ALT 3348, and LDH 4396) on postoperative day (POD) #1. Fibrinogen nadir and Hemoglobin nadir were 170 and 8.1, respectively, on POD #2. Labs were improving by POD #3, and she was discharged home. Her symptoms improved immediately postpartum, other than dysphagia. At follow-up, the patient’s incision was healing well, and she was coping appropriately.

### CONCLUSION

Similar to reports implicating COVID-19 in HELLP, we suspect a similar inflammatory process from the Influenza B diagnosis likely ignited a systemic response in our patient. This case underscores the complexity of managing pregnant patients with influenza-associated complications, highlighting the importance of prompt intervention, intraoperative considerations, and multidisciplinary care.

